# Representativeness of European clinical trial populations in mild Alzheimer’s disease dementia: a comparison of 18-month outcomes with real-world data from the GERAS observational study

**DOI:** 10.1186/s13195-018-0360-4

**Published:** 2018-04-03

**Authors:** Catherine Reed, Mark Belger, Grazia Dell’Agnello, Kristin Kahle-Wrobleski, Gopalan Sethuraman, Ann Hake, Joel Raskin, David Henley

**Affiliations:** 1 Eli Lilly and Company Limited, Erl Wood Manor, Sunninghill Road, Windlesham, Surrey, GU20 6PH UK; 2 Eli Lilly Italia, Sesto Fiorentino, Italy; 3 Eli Lilly and Company, Indianapolis, IN USA; 4 Indiana University School of Medicine, Indianapolis, IN USA

**Keywords:** Alzheimer’s disease, Randomised controlled trials, Observational studies, Real-world data, Regional differences

## Abstract

**Background:**

Comparison of disease progression between placebo-group patients from randomised controlled trials (RCTs) and real-world patients can aid in assessing the generalisability of RCT outcomes. This analysis compared outcomes between community-dwelling patients with mild Alzheimer’s disease (AD) dementia from two RCTs (pooled European (EU) data from EXPEDITION and EXPEDITION 2) and similar patients from the EU GERAS observational study.

**Methods:**

Data from placebo-group patients with mild AD dementia from the RCTs (EU countries only) were compared with data from GERAS patients with mild AD dementia. Between-group differences for changes over 18 months were analysed for cognition, functioning, neuropsychiatric symptoms, health-related quality of life (HRQoL) and caregiver time using propensity score-adjusted models. A sensitivity analysis compared EU/North American (EU/NA) EXPEDITION patients with GERAS patients.

**Results:**

EU EXPEDITION patients (*n* = 168) were younger than GERAS patients (*n* = 566) (mean (standard deviation, SD) age 71.9 (7.4) versus 77.3 (6.9) years; *p* < 0.001) and were more likely to use AD treatment (95% versus 84%; *p* < 0.001). Cognitive performance was similar at baseline in both populations, although GERAS patients showed greater functional impairment (*p* = 0.005) and lower HRQoL (*p* < 0.05). At 18 months, no statistically significant differences between EXPEDITION (*n* = 133) and GERAS (*n* = 417) patients were observed for changes in cognitive, functional, neuropsychiatric and HRQoL outcomes. Least squares mean (95% confidence interval) change in caregiver time (hours/month) spent on instrumental activities of daily living (iADL; 29.22 (19.16, 39.27) versus 3.20 (−11.89, 18.28), *p* = 0.001) and supervision (66.59 (47.49, 85.69) versus 3.04 (−25.39, 31.48), *p* < 0.001) showed greater increases in GERAS than EXPEDITION. In the sensitivity analysis, changes in neuropsychiatric and HRQoL scores and caregiver time spent on basic ADL were also significantly greater in GERAS than in EU/NA EXPEDITION patients.

**Conclusions:**

Patients with mild AD dementia participating in the EU EXPEDITION RCTs and the GERAS observational study showed a similar decline in cognitive, functional and neuropsychiatric symptoms over 18 months, whereas changes in caregiver time measures were significantly greater in GERAS. Results indicate the importance of using similar regions when comparing real-world and RCT data.

**Trial registration:**

ClinicalTrials.gov NCT00905372 EXPEDITION. Registered 18 May 2009.

ClinicalTrials.gov NCT00904683 EXPEDITION 2. Registered 18 May 2009.

## Background

Randomised controlled trials (RCTs) are the gold standard for assessing treatment efficacy [1] and are designed with internal validity as a priority. Although internal validity is important in a trial setting, RCTs are criticised for their lack of external validity or generalisability [2]. The key issue with respect to this is that trial populations are recruited using extensive exclusion criteria, which aim to select a homogeneous population with few comorbidities; this does not always represent the more heterogeneous patient populations (potentially with many comorbidities) in clinical practice. There may also be differences between RCT protocols and routine practice (e.g. diagnostic methods, treatments used and timing of treatment) and the setting of the trial (e.g. country, healthcare system or primary versus secondary care), all of which can influence external validity [2].

The outcomes measured in RCTs therefore do not always represent those considered most important in clinical practice, leaving clinicians to extrapolate the results of a trial to the ‘real world’. By contrast, observational studies can obtain data on variables that cannot be measured during RCTs, such as cost and resource use in routine clinical practice, providing greater external validity.

Comparison of disease progression and health outcomes between control arms of RCTs and real-world patient cohorts can aid in assessing the generalisability and extrapolation of RCT data to the real world. To best analyse the complementary data from RCTs and observational studies, it is important to identify both similarities and differences between patients participating in RCTs and those in real-world clinical practice. For example, as reported from multi-national RCTs, the progression of Alzheimer’s disease (AD) or its measurement can differ across geographic regions, partly due to heterogeneity across populations at baseline [3, 4]. Country- and culture-specific variations in practice patterns and healthcare systems may also be important when performing such assessments; it would be expected that data from similar countries (i.e. with similar cultures, practice patterns and healthcare systems) should therefore be used when comparing RCT and real-world data.

The GERAS observational study [5] of community-dwelling patients with AD dementia in three European (EU) countries (France, Germany and the United Kingdom (UK)) provides an ideal real-world data source with which to compare data from placebo-treated patients with AD dementia in RCTs. The analysis reported here compared 18-month disease and health outcomes between patients with probable mild AD dementia from the placebo arms of the EU populations from two RCTs (pooled data from EXPEDITION and EXPEDITION 2 [6, 7]) and those from the GERAS observational study, after adjusting for baseline differences.

## Methods

### Study designs

#### EXPEDITION programme

The EXPEDITION trials (EXPEDITION and EXPEDITION 2; ClinicalTrials.gov NCT00905372 and NCT00904683, respectively) were two 18-month, randomised, double-blind, placebo-controlled, phase III registration trials [6].

Patients participating in the trials were aged ≥ 55 years, had been diagnosed with probable AD dementia (according to National Institute of Neurological and Communicative Disorders and Stroke and Alzheimer’s Disease and Related Disorders Association (NINCDS-ADRDA) criteria [8]), lived in the community (i.e. were not institutionalised), were receiving standard-of-care treatment and had a caregiver who spent at least 10 hours/week with the patient and could attend each visit with the patient throughout the study [4]. Patients had a Mini-Mental State Examination (MMSE) score [9] of 16–26; patients with mild AD dementia were pre-specified to be those with an MMSE score of 20–26. Patients with a current serious or unstable illness were excluded, as were those with vascular dementia [4]. Patients receiving concomitant treatment with an acetylcholinesterase inhibitor (AChEI) or memantine had to have been on the medication for ≥ 4 months with a stable dose for ≥ 2 months.

Patients were recruited from Europe, North America, South America, Asia and Australia between May 2009 and December 2010. The study protocol was approved by the institutional review board at each participating institution, and all participants provided written informed consent. Data from all patients with mild AD dementia in the EXPEDITION and EXPEDITION 2 trials were pooled to maximise the sample size. To minimise differences due to region-specific variations, but to allow sufficient patient numbers for comparison purposes, only data from EU populations (i.e. France, Germany, Italy, Poland, Spain, Sweden and the UK) in the EXPEDITION RCTs were used in the current analysis.

#### GERAS study

GERAS was an 18-month, prospective, observational study of patients with probable AD dementia of all severities who presented within the course of normal clinical care in France, Germany and the UK [5], with the main objective of estimating the societal cost of AD dementia in these countries.

The study included community-dwelling patients aged ≥ 55 years who had been diagnosed with probable AD dementia (according to NINCDS-ADRDA criteria) and had an MMSE score of ≤ 26 and an informal (i.e. non-professional) caregiver who was responsible for the patient for at least 6 months of the year. Patients with Parkinson’s disease at or before AD onset or probable Lewy body disease and those participating in an interventional study were excluded. AD treatment could be prescribed according to standards of care during the study, and treatment decisions were at the discretion of the physician and patient. Patients included in the present analyses were those with mild AD dementia (MMSE score of 21–26 [10]) at the time of enrolment in the GERAS study.

Patients were enrolled between October 2010 and September 2011. The study was approved by ethical review boards in accordance with country-specific regulations; written informed consent was obtained from all participants or their legal representative.

### Data collected

Data collected in all studies included patient and caregiver demographics and patient clinical characteristics at baseline, such as comorbidities and medication use, cognitive function, ability to perform basic and instrumental activities of daily living (bADL and iADL), neuropsychiatric symptoms and health-related quality of life (HRQoL) and caregiver time (hours/month) spent on bADL, iADL and supervision. Due to the differing nature of the GERAS and EXPEDITION studies, some assessments were performed more frequently in the EXPEDITION trials than in the GERAS study. The analysis reported here therefore focuses on those time points common to all studies.

Cognitive function was assessed using both the MMSE [9] and the 14-item cognitive subscale of the Alzheimer’s Disease Assessment Scale (ADAS-Cog14 [11, 12]). Poorer cognition is indicated by lower MMSE scores and higher ADAS-Cog14 scores (total MMSE score range 0–30 and ADAS-Cog14 score range 0–90). MMSE data at baseline and at 6, 12 and 18 months, and ADAS-Cog14 data at baseline and 18 months, were included in this analysis.

The patient’s functional ability (i.e. ability to perform bADL and iADL) was assessed according to the Alzheimer’s Disease Cooperative Study Activities of Daily Living Inventory (ADCS-ADL [13]). Poorer functioning is indicated by lower scores; possible scores range from 0 to 22 (bADL) and from 0 to 56 (iADL). Baseline and 18-month data were included in this analysis.

Neuropsychiatric symptoms were recorded according to the Neuropsychiatric Inventory (NPI)-12 [14, 15]. Scores range from 0 to 144, with higher scores indicating poorer neuropsychiatric function. The NPI-12 Caregiver Distress Score [15] was also recorded; scores range from 0 to 60, with higher scores indicating greater caregiver distress. NPI-12 data at baseline and at 6, 12 and 18 months were included in this analysis.

HRQoL was measured using the EuroQoL-5-Dimensions questionnaire (EQ-5D [16]); caregivers completed the proxy version on behalf of the patient. Both UK population-based index scores and visual analogue scale (VAS) scores were recorded. Index scores range from 0 to 1, and VAS scores range from 0 to 100; lower scores indicate reduced HRQoL on each of these measures. EQ-5D index and VAS scores at baseline and 18 months were included in this analysis.

Data on caregiver time (hours/month) spent on bADL, iADL and supervision were obtained using the Resource Utilization in Dementia (RUD)-Lite instrument [17] in the EXPEDITION and EXPEDITION 2 trials and using the standard RUD instrument [17] in the GERAS study. Data (for the month prior to data collection) at baseline and at 6, 12 and 18 months were included in this analysis.

### Statistical analysis

This analysis compared a pooled group of patients with mild AD dementia at baseline from the placebo arms of the EXPEDITION and EXPEDITION 2 RCTs (EU EXPEDITION populations only) and patients with mild AD dementia at baseline from the GERAS study.

Demographics and baseline characteristics were summarised using descriptive statistics based on non-missing observations. Differences in baseline measures between study type were tested using analysis of variance (ANOVA) for continuous measures and the Cochran–Mantel–Haenszel test for categorical measures. However, the Mann–Whitney test was used for caregiver time because of the skewed distribution of the data.

Propensity scores [18] were calculated to account for differences in baseline patient characteristics between EXPEDITION and GERAS, including differences in the lower cut-off of MMSE score used to define mild AD dementia at baseline (mild AD dementia was classified as MMSE score 20–26 in EXPEDITION but 21–26 in GERAS). The propensity score included the following variables: patient age, sex, number of comorbidities, time since diagnosis of AD dementia, years of education, use of AD medication (yes/no) and baseline MMSE score. Absolute standardised differences [19] were calculated for baseline covariates before and after propensity score adjustment; a difference of < 0.1 was considered an acceptable balance between the covariates.

Differences between study types for changes in outcomes over 18 months were analysed using repeated-measures models where more than one post-baseline visit was recorded, or analysis of covariance (ANCOVA) otherwise. Models included the following covariates: patient age, patient receiving AD medication (yes/no), propensity score and baseline outcome score. Study type, visit and study type × visit interaction were fitted for repeated-measures models, whereas only study type was included for outcomes with just one common post-baseline visit (i.e. ADAS-Cog14, ADCS-ADL and EQ-5D). Data from the models were reported as least squares (LS) means.

A more rigorous set of propensity scores was calculated by including all common baseline patient and caregiver characteristics and their two-way interactions, where the majority of baseline characteristics achieved an absolute standardised difference of < 0.1.

Sensitivity analyses were performed to assess the effect of including EU and North American (EU/NA) patients from the EXPEDITION RCTs and the full EXPEDITION population in the comparison with GERAS patients.

Analyses were conducted using SAS software version 9.2 (SAS Institute, Cary, NC, USA).

## Results

### Baseline characteristics

A total of 168 EU EXPEDITION patients and 566 GERAS patients and their caregivers were included in this analysis at baseline (Table 1).

**Table 1:** Baseline patient and caregiver characteristics

Characteristic	EXPEDITION RCTs (EU population)	GERAS observational study	*p* value for unadjusted baseline comparison
Patients, *N*	168	566	
Age (years)	71.9 (7.4)	77.3 (6.9)	**< 0.001**
Sex (female), *n* (%)	85 (50.6)	271 (47.9)	0.83
Patient education (years)	11.6 (3.9)	11.1 (3.3)	0.06
Time since AD diagnosis (years)	1.7 (1.5)	1.7 (2.0)	0.90
AD treatment category, *n* (%)			**< 0.001**
AChEI only	129 (76.8)	411 (72.6)	
Memantine only	12 (7.1)	41 (7.2)	
AChEI + memantine	19 (11.3)	26 (4.6)	
No AD treatment	8 (4.8)	88 (15.5)	
MMSE score	23.1 (2.0)	23.3 (1.6)	0.15
Caregivers, *N*	168	566	
Age (years)	64.2 (12.2)	68.1 (11.6)	**< 0.001**
Sex (female), *n* (%)	101 (60.5)	387 (68.5)	0.21
Caregiver is patient’s spouse, *n* (%)	116 (69.5)	399 (70.6)	0.82
Caregiver is working for pay, *n* (%)	57 (34.1)	133 (23.5)	0.04

Data presented as mean (standard deviation) unless otherwise indicated. Percentages based on the number of patients/caregivers with available data (missing data ranged from zero to one participant in the EXPEDITION trials and from zero to three participants in the GERAS study). *p*-values in bold represent a significant difference between EXPEDITION and GERAS

*AChEI* acetylcholinesterase inhibitor, *AD* Alzheimer’s disease, *EU* European, *MMSE* Mini-Mental State Examination, *RCT* randomised controlled trial

EXPEDITION patients were younger than those in the GERAS study (mean (standard deviation, SD) age 71.9 (7.4) versus 77.3 (6.9) years, respectively; *p* < 0.001) and showed greater use of any AD treatment (at least one AChEI and/or memantine) and combination AD treatment (AChEI + memantine; *p* < 0.001 for differences in AD treatment category between the groups) (Table 1). No significant differences were found for other patient baseline characteristics.

Caregivers of subjects in the EXPEDITION trials were younger than those in the GERAS study (mean (SD) age 64.2 (12.2) versus 68.1 (11.6) years, respectively; *p* < 0.001).

Baseline cognition measures (MMSE and ADAS-Cog14 scores) were similar between the groups (Tables 1 and 2), although GERAS patients showed slightly greater functional impairment (*p* = 0.005 for bADL, *p* < 0.001 for iADL) and lower HRQoL (*p* < 0.001 for EQ-5D index scores, *p* = 0.03 for VAS scores) but slightly (although not statistically significant) less neuropsychiatric impairment (*p* = 0.07).

**Table 2:** Baseline patient and caregiver outcomes

Outcome	EXPEDITION RCTs (EU population)	GERAS observational study	*p* value for unadjusted baseline comparison
Patients, *N*	168	566	
ADAS-Cog14 score (range 0–90 [11,12])	29.4 (8.6)	30.4 (7.8)	0.16
ADCS-ADL basic score (range 0–22 [13])	20.6 (2.5)	19.8 (3.1)	**0.005**
ADCS-ADL instrumental score (range 0–56 [13])	42.5 (9.9)	38.5 (11.8)	**< 0.001**
NPI-12 total score (range 0–144 [14,15])	12.0 (12.8)	10.2 (10.7)	0.07
EQ-5D UK population-based index score (range 0–1 [16])	0.79 (0.18)	0.71 (0.24)	**< 0.001**
EQ-5D VAS score (range 0–100 [16])	62.2 (24.1)	66.0 (18.5)	0.03
Caregivers, *N*	168	566	
NPI-12 Caregiver Distress score (range 0–60 [15])	7.1 (7.1)	6.4 (6.2)	0.26
Caregiver time spent on patient (hours in past month)			
Basic ADL, mean (SD)	9.8 (26.9)	16.4 (44.7)	**0.009**
Median (IQR)	0 (0–0)	0 (0–15)	
Instrumental ADL, mean (SD)	53.5 (61.9)	61.0 (83.1)	0.23
Median (IQR)	30 (2–90)	36 (8–90)	
Supervision, mean (SD)	24.9 (89.1)	48.4 (123.6)	**< 0.001**
Median (IQR)	0 (0–0)	0 (0–30)	
Overall, mean (SD)	86.3 (128.5)	121.6 (174.2)	**0.01**
Median (IQR)	30.6 (3–120)	60 (15–125)	

Data presented as mean (standard deviation) unless stated otherwise. Missing data ranged from one to three participants in the EXPEDITION trials and from one to eight participants in the GERAS study. *p*-values in bold represent a significant difference between EXPEDITION and GERAS

Poorer cognition is indicated by higher scores for ADAS-Cog14; poorer functioning is indicated by lower ADCS-ADL basic and instrumental scores; poorer neuropsychiatric function is indicated by higher NPI-12 scores; reduced HRQoL is indicated by lower EQ-5D scores; greater caregiver distress is indicated by higher NPI-12 Caregiver Distress scores

*ADAS-Cog14* 14-item cognitive subscale of the Alzheimer’s Disease Assessment Scale, *ADL* activities of daily living, *ADCS-ADL* Alzheimer’s Disease Cooperative Study Activities of Daily Living Inventory, *EQ-5D* EuroQoL-5-Dimensions questionnaire, *EU* European, *HRQoL* health-related quality of life, *IQR* interquartile range, *NPI* Neuropsychiatric Inventory, *RCT* randomised controlled trial, *SD* standard deviation, *UK* United Kingdom, *VAS* visual analogue scale

All caregiver time measures (hours in past month) except for time spent on iADL were significantly greater in GERAS than in EXPEDITION at baseline (*p* ≤ 0.01; Table 2).

**Fig. 1:**
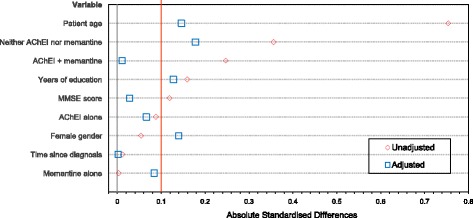
Standardised differences (between EU EXPEDITION and GERAS patients) for baseline characteristics before and after propensity score adjustment. *AChEI* acetylcholinesterase inhibitor, *MMSE* Mini-Mental State Examination

The propensity score adjustments resulted in no significant differences between the baseline characteristics of EU EXPEDITION and GERAS patients, although not all of the baseline characteristics achieved the recommended absolute standardised difference of < 0.1 (Fig. 1).

The alternative propensity score approaches gave similar results to the primary analysis for cognition, functioning, neuropsychiatric symptoms and caregiver time (data not shown).

### Comparison of 18-month outcomes between EU EXPEDITION and GERAS

**Fig. 2:**
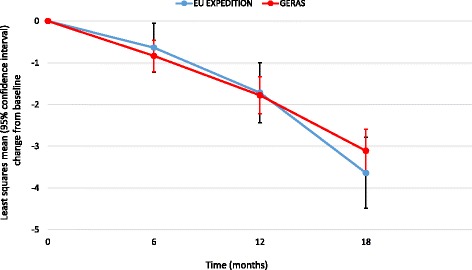
Change in Mini-Mental State Examination score (propensity score-adjusted). *p* = 0.7912 for overall difference between study type; *p* = 0.1297 for study type × visit interaction. *EU* European

**Fig. 3:**
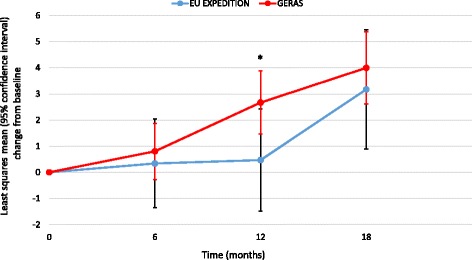
Change in Neuropsychiatric Inventory-12 score (propensity score-adjusted). *p < 0.05 for difference between study type at 12 months; *p* = 0.1882 for overall difference between study type; *p* = 0.1580 for study type × visit interaction. *EU* European

Eighteen-month data were available for 133 EXPEDITION patients (79%) and 417 GERAS patients (74%). At 18 months, no statistically significant differences between the two groups were observed for changes in cognitive, functional, neuropsychiatric or HRQoL outcomes (Figs. 2 and 3; Table 3), although the change in NPI-12 score was significantly higher in GERAS than in EXPEDITION caregivers at 12 months (Fig. 3).

**Table 3:** Change from baseline in patient and caregiver outcomes at 18 months (propensity score-adjusted)

Outcome	LS mean (95% CI) change from baseline		Difference (95% CI)	ANCOVA *p* value for study type
	EXPEDITION RCTs (EU population)	GERAS observational study		
ADAS-Cog14 score	6.73 (4.68, 8.79)	4.92 (3.49, 6.35)	1.81 (−0.30, 3.92)	0.09
ADCS-ADL basic score	−1.80 (−2.54, −1.06)	−1.80 (−2.29, −1.30)	−0.00 (−0.76, 0.75)	0.99
ADCS-ADL instrumental score	−7.42 (−9.45, −5.39)	−7.29 (−8.64, −5.94)	−0.13 (−2.22, 1.96)	0.90
EQ-5D UK population-based index score	−0.036 (−0.087, 0.015)	−0.069 (−0.103, −0.034)	0.032 (−0.020, 0.085)	0.22
EQ-5D VAS score	−0.60 (−4.41, 3.21)	−2.11 (−4.92, 0.70)	1.51 (−2.42, 5.44)	0.45
NPI-12 Caregiver Distress score	1.79 (0.51, 3.07)	1.90 (1.13, 2.68)	−0.11 (−1.49, 1.27)	0.90

Poorer cognition is indicated by higher scores for ADAS-Cog14; poorer functioning is indicated by lower ADCS-ADL basic and instrumental scores; reduced HRQoL is indicated by lower EQ-5D scores; greater caregiver distress is indicated by higher NPI-12 Caregiver Distress scores

*ADAS-Cog14* 14-item cognitive subscale of the Alzheimer’s Disease Assessment Scale, *ADCS-ADL* Alzheimer’s Disease Cooperative Study Activities of Daily Living Inventory, *ANCOVA* analysis of covariance, *CI* confidence interval, *EQ-5D* EuroQoL-5-Dimensions questionnaire, *EU* European, *LS* least squares, *MMSE* Mini-Mental State Examination, *NPI* Neuropsychiatric Inventory, *RCT* randomised controlled trial, *UK* United Kingdom, *VAS* visual analogue scale

Change in caregiver time (hours/month) spent on bADL did not differ significantly between the two groups (*p* = 0.14 for difference between study type).

**Fig. 4:**
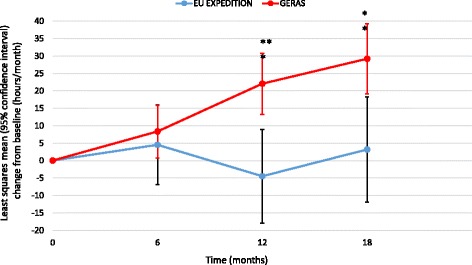
Change in caregiver time spent on instrumental activities of daily living (propensity score-adjusted). **p < 0.01; ***p < 0.001 for difference between study type at that time point; *p* = 0.0011 for overall difference between study type; *p* = 0.0023 for study type × visit interaction. *EU* European

**Fig. 5:**
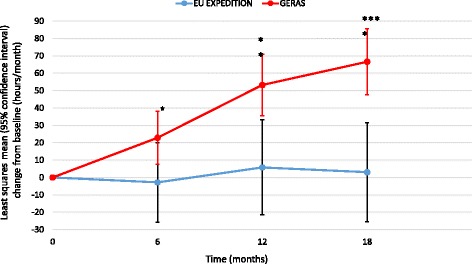
Change in supervision time (propensity score-adjusted). *p < 0.05; **p < 0.01; ****p < 0.0001 for difference between study type at that time point; *p* = 0.0001 for overall difference between study type; *p* = 0.0254 for study type × visit interaction. *EU* European

**Fig. 6:**
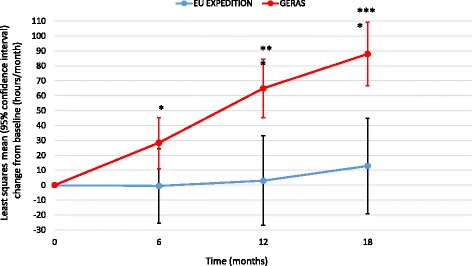
Change in overall caregiver time (propensity score-adjusted). *p < 0.05; ***p < 0.001; ****p < 0.0001 for difference between study type at that time point; *p* < 0.0001 for overall difference between study type; *p* = 0.0074 for study type × visit interaction. *EU* European

All other caregiver time measures showed greater increases over 18 months in the GERAS versus EXPEDITION populations (*p* = 0.001 for time spent on iADL; *p* < 0.001 for supervision time and overall caregiver time; Figs. 4, 5 and 6).

### Sensitivity analysis

Sensitivity analysis results based on comparing the EU/NA EXPEDITION population (*N* = 466) with GERAS patients (*N* = 566) were similar to those based on the EU-only EXPEDITION population in terms of cognitive, functioning and time measures. Changes in NPI-12, NPI caregiver distress and EQ-5D scores and caregiver time spent on bADL were also significantly greater for GERAS than for EU/NA EXPEDITION patients (Table 4). When the full EXPEDITION population (*N* = 663) from all geographic regions was compared with the GERAS population (*N* = 566), differences were also observed between ADCS-ADL basic and instrumental scores, but the difference in caregiver time spent on bADL based on the EU/NA EXPEDITION population was not seen for the full population analysis (Table 4).

**Table 4:** Sensitivity analysis: differences between EXPEDITION and GERAS in change from baseline in propensity score-adjusted outcomes

	Difference versus GERAS (95% CI)			
Outcome	EU EXPEDITION	EU/NA EXPEDITION	All EXPEDITION	Population(s) showing significant difference
MMSE total score	−0.53 (−1.46, 0.40)	0.06 (−0.62, 0.74)	0.40 (−0.20, 1.00)	NS
ADAS-Cog14 score	1.81 (−0.30, 3.92)	0.66 (−1.06, 2.39)	−0.36 (−1.85, 1.13)	NS
ADCS-ADL basic score	−0.003 (−0.76, 0.75)	0.41 (−0.16, 0.98)	**0.67** **(0.20, 1.15)**	**Full only** ^**a**^
ADCS-ADL instrumental score	−0.13 (−2.22, 1.96)	1.51 (−0.11, 3.12)	**2.42** **(1.02, 3.82)**	**Full only** ^**a**^
EQ-5D UK population-based index score	0.03 (−0.02, 0.08)	**0.06** **(0.02, 0.10)**	**0.07** **(0.03, 0.10)**	**Full, EU/NA** ^**b**^
EQ-5D VAS score	1.51 (−2.42, 5.44)	**4.66** **(1.69, 7.63)**	**4.63** **(2.01, 7.25)**	**Full, EU/NA** ^**b**^
NPI-12 total score	−0.82 (−3.28, 1.63)	**−2.36** **(−4.17, −0.56)**	**−3.11** **(−4.67, −1.55)**	**Full, EU/NA** ^**b**^
NPI-12 Caregiver Distress score	−0.11 (−1.49, 1.27)	**−1.22** **(−2.21, −0.24)**	**−1.64** **(−2.51, −0.78)**	**Full, EU/NA** ^**b**^
Caregiver time on basic ADL (hours/month)	−9.41 (−23.81, 4.99)	**−12.27** **(−22.91, −1.63)**	−9.69 (−19.66, 0.28)	**EU/NA only** ^**c**^
Caregiver time on instrumental ADL (hours/month)	**−26.02** **(−42.86, −9.18)**	**−20.44** **(−34.16, −6.72)**	**−21.96** **(−34.52, −9.40)**	**All** ^**d**^
Supervision time (hours/month)	**−63.55** **(−94.89, −32.22)**	**−57.33** **(−79.81, −34.86)**	**−57.46** **(−77.09, −37.83)**	**All** ^**d**^
Overall caregiver time (hours/month)	**−75.01** **(−110.37, −39.64)**	**−71.67** **(−98.28, −45.06)**	**−76.33** **(−100.07, −52.58)**	**All** ^**d**^

Data presented as least squares mean difference (95% CI). Results in bold are significantly different for EXPEDITION versus GERAS populations (*p* < 0.05)

*ADAS-Cog14* 14-item cognitive subscale of the Alzheimer’s Disease Assessment Scale, *ADCS-ADL* Alzheimer’s Disease Cooperative Study Activities of Daily Living Inventory, *ADL* activities of daily living, *CI* confidence interval, *EQ-5D* EuroQoL-5-Dimensions questionnaire, *EU* European, *EU/NA* European/North American, *MMSE* Mini-Mental State Examination, *NPI* Neuropsychiatric Inventory, *NS* No significant differences at 18 months in any analysis, *UK* United Kingdom, *VAS* visual analogue scale

^a^Greater impairment in GERAS based on the full EXPEDITION population only

^b^Greater impairment in GERAS based on the full EXPEDITION and EU/NA populations only

^c^Greater impairment in GERAS based on EU/NA population only

^d^Greater impairment in GERAS for all populations

Propensity score adjustment achieved standardised differences of < 0.1 for all variables based on the EU/NA EXPEDITION and full EXPEDITION populations (data not shown).

## Discussion

Our analysis showed similar results between RCT and real-world data for changes in cognitive, functional and neuropsychiatric symptoms over 18 months in patients with mild AD dementia, after controlling for baseline differences.

Significant differences in NPI-12 and EQ-5D score changes were identified in our sensitivity analysis when more diverse regions (EU/NA) were included in the EXPEDITION cohort. When the full EXPEDITION cohorts were included, small but significant baseline-adjusted differences between the studies were observed in the change in functional and neuropsychiatric symptoms at 18 months, as reported in our previous analysis [20]. The lack of such differences when EU-only patients from EXPEDITION were compared with GERAS patients suggests that these differences may have been due to multi-country or culture-specific variations rather than to differences in study design and indicate the importance of using geographically similar regions when comparing real-world data with RCT results to better reflect healthcare systems and/or socio-economic similarities.

Controlling for unmeasured confounding (e.g. differences in AD diagnosis and standard-of-care treatment within healthcare systems) is an issue when studies cover wide geographical regions. Our data suggest that RCT clinical and health outcomes can be generalised to real-world populations (adjusting for known baseline differences) within comparable geographical regions and reinforce recommendations that clinical trial designs should consider the heterogeneity of global populations when planning country and regional participation [3, 4].

Significant differences between the EXPEDITION and GERAS studies were observed for changes in all measures of caregiver time, except for time spent on bADL. The lack of difference in time spent on bADL may be due to the relatively small amount of time spent on this aspect of care; time on bADL accounted for the lowest proportion of caregiver time at baseline in both studies, which is to be expected for patients with mild AD dementia. Significant differences in changes in caregiver time were consistent between the primary and sensitivity analyses; although the change in caregiver time spent on bADL was statistically significantly different between the EU/NA EXPEDITION population and GERAS patients, this numerically small difference is unlikely to be of clinical significance.

Caregiver time spent on iADL and supervision, and overall caregiver time, all showed greater increases over 18 months in the GERAS than in the EXPEDITION populations. This difference was particularly apparent for supervision time. Little change in caregiver time was observed over 18 months in the EXPEDITION population, whereas an increase over time was seen in GERAS; this difference in trends could not be controlled for in the models. This may reflect a selection bias for patients in the RCT cohort who are more independent and thus better candidates for participation in a complex clinical trial.

Although baseline functioning was lower and caregiver time measures (except for time spent on iADL) were higher in GERAS than EXPEDITION, these differences were adjusted for in the models for each outcome of interest. Despite controlling for several baseline factors, it is possible that unmeasured confounders may have contributed to our findings. In addition, it is important to consider that patient populations generally differ between RCTs and observational studies due to the RCT setting of highly specialised academic research clinics versus more regional or local memory clinics in observational studies. Although differences in baseline data were controlled for, patients in GERAS were older than those in the EXPEDITION trials and fewer caregivers were working for pay. It is therefore likely that the patient population in the RCTs was healthier (possibly a factor in their better functioning), which may have contributed to the requirement for less caregiver time than in GERAS. As comorbidity data were collected differently in the two study types, distinguishing between comorbidities was not possible with the available data; we therefore used a high-level indicator of comorbidities (the mean number of comorbidities from 10 specific conditions) for the purpose of measuring the general health of both populations for propensity scoring. In GERAS, patients with mild AD dementia had a mean (SD) of 1.5 (1.2) comorbidities at baseline, based on 10 specific self-reported comorbidities. These were included in the propensity score along with similar comorbidities reported from the EXPEDITION populations (estimated mean (SD) 1.2 (1.2)).

Other factors which may influence caregiver time are that the type of caregivers who are motivated to participate in RCTs may also be more able to utilise available resources within the study framework and elsewhere to manage caregiving time effectively. Caregiver definitions also differed between the studies; the caregiver in the EXPEDITION cohort could be a professional or informal caregiver who spent at least 10 hours/week with the patient, whereas GERAS data were based on the informal primary caregiver. Although both populations had a similar proportion of spousal caregivers (~ 70%), it is possible that increasing ADL needs were met by additional professional caregivers in the EXPEDITION trials. In addition, 53–57% of GERAS caregivers were sole caregivers over the 18-month period, but this information was not collected in the EXPEDITION trials.

Strengths of our analysis include the comparison of RCT and real-world data from geographically similar populations (as recommended by Henley et al. [3] and Grill et al. [4]), thus limiting confounders due to global diversity in patient/caregiver cultural factors and variations in health and social care provision. The models controlled for differences in baseline characteristics and outcomes to limit any influence of known baseline variations.

This analysis is also subject to some limitations. First, although the populations with mild AD dementia only were compared between EXPEDITION and GERAS, the criteria for mild AD dementia differed slightly between the studies (MMSE score 20–26 in EXPEDITION, 21–26 in GERAS). However, we do not believe that this small difference influenced our results, as baseline MMSE scores were included in the propensity scoring. Second, as mentioned previously, although few significant differences were observed between baseline characteristics, propensity score adjustment did not achieve standardised differences < 0.1 for all variables, including patient age, suggesting that some confounding may not have been accounted for. As it was not possible to distinguish between different types of comorbidities in the different studies, this may have been a contributing factor limiting the propensity score. Third, the adjustment of outcomes by propensity score was used to determine whether the progression of AD dementia was similar between a RCT cohort and an observational cohort; when disease progression is similar after adjustment for differences in patient characteristics, it would suggest that patients enrolled in an observational study like GERAS are similar to those enrolled in a RCT if they have the same baseline characteristics. By using a propensity score approach, differences between the populations are not taken into account when comparing disease progression. However, similar trajectories of disease progression following adjustment of baseline characteristics would suggest that unmeasured differences between the patient characteristics are not influencing disease trajectory. Fourth, all potential confounders that may have impacted on outcomes were not captured by both studies (e.g. apolipoprotein E4) and therefore could not be included in this analysis. Unmeasured confounders, including potential Hawthorne effects in RCTs and observational studies, were not included in this analysis. Fifth, the type of study site was not controlled for in the analysis; as study centres in the GERAS study were less specialised/research oriented than those in the RCTs, it is likely that levels of training and experience with the scales used to assess patients varied between the study programmes and centres. Last, additional countries were included in the RCT analysis group in order to have a sufficient sample size for comparisons with the results from the three countries where GERAS data were collected. Although differences in healthcare practices and treatment patterns between those countries included and not included in the GERAS study are likely, our results found consistent disease progression between patients participating in the RCTs or the observational study. However, given that the optimal comparison for this research question would be to use data from the same countries for both the GERAS study and the RCTs, we are cautious in stating that these findings are based on regional, rather than country-specific, findings.

## Conclusions

Using a propensity score matching approach, a similar decline in cognition and functioning was observed between results from RCT and observational study patients in the EU population. Our findings support that RCTs and observational studies can provide complementary data to assess longitudinal disease progression in patients with mild AD dementia. Confounding factors inherent within the different study designs and inclusion criteria mean that some findings require additional explanation; for example, the increase in caregiver time over 18 months in the GERAS study but not during the RCTs may have resulted from differences in the type of care received or baseline differences not accounted for in propensity matching. Use of similar geographic regions is important when comparing real-world and RCT data to ensure that differences between studies are not simply due to country-specific variations. Further opportunities to assess the comparability of real-world and RCT data will arise when data from additional countries in the GERAS study (USA and Japan) are available. These will help inform which parameters and outcomes are culturally/geographically dependent.

## Abbreviations

*AChEI*: Acetylcholinesterase inhibitor
*AD*: Alzheimer’s disease
*ADAS-Cog14*: 14-item cognitive subscale of the Alzheimer’s Disease Assessment Scale
*ADCS-ADL*: Alzheimer’s Disease Cooperative Study Activities of Daily Living Inventory
*ADRDA*: Alzheimer’s Disease and Related Disorders Association
*ANOVA*: Analysis of variance
*bADL*: Basic activities of daily living
*EQ-5D*: EuroQoL-5-Dimensions questionnaire
*EU*: European
*HRQoL*: Health-related quality of life
*iADL*: Instrumental activities of daily living
*LS*: Least squares
*MMSE*: Mini-Mental State Examination
*NINCDS*: National Institute of Neurological and Communicative Disorders and Stroke
*NPI*: Neuropsychiatric Inventory
*RCT*: Randomised controlled trial
*RUD*: Resource Utilization in Dementia
*SD*: Standard deviation
*UK*: United Kingdom
*VAS*: Visual analogue scale
